# Recursive Algorithms for Modeling Genomic Ancestral Origins in a Fixed Pedigree

**DOI:** 10.1534/g3.118.200340

**Published:** 2018-08-09

**Authors:** Chaozhi Zheng, Martin P. Boer, Fred A. van Eeuwijk

**Affiliations:** Biometris, Wageningen University and Research, Wageningen, The Netherlands

**Keywords:** Junction theory, Identical by descent, Ancestral inference, QTL mapping resolution, Collaborative Cross (CC), Recombinant Inbred Line (RIL), Multiparent Advanced Generation InterCross (MAGIC), multiparental populations, MPP

## Abstract

The study of gene flow in pedigrees is of strong interest for the development of quantitative trait loci (QTL) mapping methods in multiparental populations. We developed a Markovian framework for modeling ancestral origins along two homologous chromosomes within individuals in fixed pedigrees. A highly beneficial property of our method is that the size of state space depends linearly or quadratically on the number of pedigree founders, whereas this increases exponentially with pedigree size in alternative methods. To calculate the parameter values of the Markov process, we describe two novel recursive algorithms that differ with respect to the pedigree founders being assumed to be exchangeable or not. Our algorithms apply equally to autosomes and sex chromosomes, another desirable feature of our approach. We tested the accuracy of the algorithms by a million simulations on a pedigree. We demonstrated two applications of the recursive algorithms in multiparental populations: design a breeding scheme for maximizing the overall density of recombination breakpoints and thus the QTL mapping resolution, and incorporate pedigree information into hidden Markov models in ancestral inference from genotypic data; the conditional probabilities and the recombination breakpoint data resulting from ancestral inference can facilitate follow-up QTL mapping. The results show that the generality of the recursive algorithms can greatly increase the application range of genetic analysis such as ancestral inference in multiparental populations.

Many complicated experimental crosses have recently been produced for mapping QTL, particularly in plants (*e.g.*, Kover *et al.* 2009; Sannemann *et al.* 2015). In contrast to traditional biparental populations, multiple parents have been used to increase genetic diversity and thus QTL segregation probability, and many generations of intercross mating have been used to increase accumulated recombination breakpoints in sampled offspring in the final generation and thus QTL mapping resolution. The expected density of recombination points is one of key quantities for optimizing experimental designs prior to collecting genotypic and phenotypic data (Rockman and Kruglyak 2008). Furthermore, the identification of recombination breakpoints from genotypic data provides useful information for increasing detection power and mapping resolution (Xu 2013; Li *et al.* 2015). The primary aims of this paper are to develop the theory of gene flow in a fixed pedigree with arbitrary structure to calculate the prior distribution of recombination points, and to apply the theory for ancestral inference and detection of recombination points from genotypic data in multiparental populations.

The theory of one-locus gene flow in a pedigree has been well developed. The haploid genomes of pedigree founders are designated by distinct labels, called founder genome labels (FGLs). A set of genes are identical by descent (IBD) if they carry the same FGLs. The identity state for two genes is either IBD or non-IBD. The IBD probability for two distinct genes within an individual is the inbreeding coefficient, and it is the kinship coefficient for the two genes randomly sampled from two distinct individuals. Rostron (1978) developed a recursive algorithm for calculating the two-gene coefficients of inbreeding and kinship. Karigl (1981) developed a recursive algorithm for calculating the coefficients of the fifteen gene identity states for four genes between two individuals. Thompson (1983) showed that a similar algorithm applies to the probabilities of joint descent of multiple genes from a specific FGL. Bauman *et al.* (2008) and Zhou *et al.* (2012) developed a recursive algorithm for calculating the ancestral coefficients such as the probability of one gene descending from a given FGL and the probability of two genes descending from two given FGLs (distinct or not).

The theory of gene flow at two linked loci in a pedigree has also been developed. Weir and Cockerham (1969) and Cockerham and Weir (1973) developed a recursive algorithm for calculating the two-locus inbreeding coefficient of an individual that is defined as a linear function of the probabilities of the fifteen identity states for four genes, two at each of two linked loci. Thompson (1988) developed a recursive algorithm for calculating the two-locus kinship between two individuals defined as the probability of IBD at both loci, see Hill and Weir (2007) for calculating multi-locus IBD probabilities in random mating populations. In contrast to the identity coefficients, the ancestral coefficient at two loci is defined as the two-locus diplotype probabilities, and there are $4^{L}$ possible diplotypes for four genes in an individual with respect to *L* distinct FGLs. Thus, the calculation of the two-locus ancestral inferences has been developed only for simple breeding systems including selfing, brother-sister (or sibling) mating, and parent-offspring mating (Haldane and Waddington 1931; Johannes and Colome-Tatche 2011; Broman 2012).

There has been much interest in developing the theory of gene flow in a pedigree with chromosomes assumed to be a genomic continuum. Donnelly (1983) developed a formal mathematical framework, where the crossover processes in a chromosome pedigree follow jointly a continuous time Markov random walk on the vertices of a hypercube, the time parameter being position along homologous chromosomes. Here a vertex represents one of possible gene transmissions from founders to all non-founders, and the number of vertices in a d-dimensional hypercube is $2^{d}$, *d* being the number of non-founders. This framework has been used in many kinds of small pedigree systems (*e.g.*, Bickeböller and Thompson 1996; Stefanov 2000; Martin and Hospital 2011). However, it is impractical to apply Donnelly’s Markovian framework to a large complex pedigree since the number of states (hypercube vertices) increases exponentially with the pedigree size.

Alternatively, the theory of junctions has been developed to study the gene flow for a genomic continuum (Fisher 1949, 1954). A junction is defined as a boundary point (recombination breakpoint) on a chromosome where two distinct FGLs meet. Fisher (1949, 1954) and Bennett (1953, 1954) developed the theory of junctions for simple breeding systems including selfing, brother-sister mating, and parent-offspring mating. It has been extended to populations (Stam 1980; Baird *et al.* 2003; Chapman and Thompson 2003; MacLeod *et al.* 2005; Zheng *et al.* 2014; Zheng 2015). In particular, Zheng *et al.* (2014) and Zheng (2015) developed a Markovian framework for modeling ancestral origins within an individual with the state space being the possible pairs of FGLs, and thus the number of states is much smaller than that of Donnelly’s Markovian framework (Donnelly 1983). On the other hand, our Markovian framework (Zheng *et al.* 2014; Zheng 2015) is restricted by two assumptions: the mating scheme from one generation to the next is random mating, and the FGLs are assumed to be exchangeable so that the probability distribution of ancestral origins in offspring is invariant to all possible permutations of the FGLs. However, the exchangeability generally does not hold for a mapping population produced via an arbitrary breeding pedigree.

In this paper, we relax the restriction of our previous Markovian framework by extending it to a fixed pedigree. We first describe a recursive algorithm (denoted by EXCH) under the assumption of FGLs being exchangeable, which is applicable for the construction of Markovian framework in simple breeding schemes. Then we develop a recursive algorithm (denoted by NON-EXCH) for modeling ancestral origins to relax the exchangeability assumption. The two recursive algorithms apply to both autosomes and sex chromosomes if they exist. The results of both recursive algorithms are compared with those from extensive simulations on a classical pedigree. We first apply the two algorithms to compare different population designs (or breeding pedigrees) prior to experiments, for example, in terms of the overall density of junctions (recombination breakpoints), an important factor of QTL mapping resolution. In addition, the non-exchangeability can be illustrated under various breeding designs. Then we apply the two algorithms to incorporate pedigree information for ancestral inference from genotypic data in simulated and real collaborative cross (CC) populations, resulting in conditional probabilities that are necessary for downstream QTL mapping.

## Recursive Algorithm Exch

### Notations and overview

**Figure 1:**
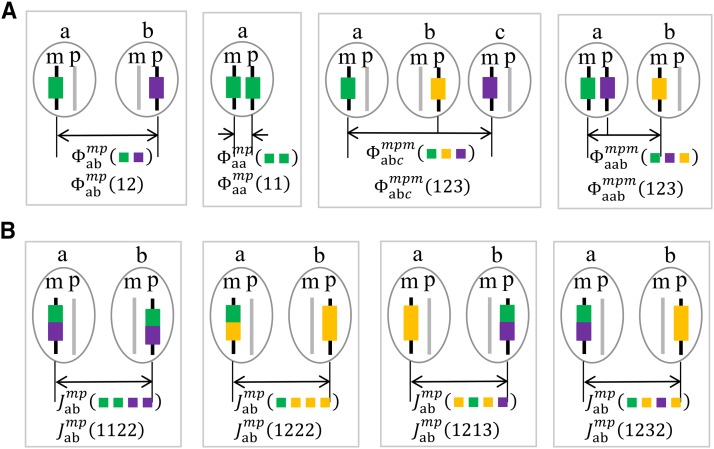
Illustration of some quantities. An identity state may correspond to many ancestral states. Different FGLs are shown by different colors, and the irrelevant chromosomes are shown as gray. (A) Some two- or three-gene ancestral coefficients and their corresponding identity coefficients. (B) Some expected ancestral junction densities and their corresponding expected identity junction densities. See Table 1 for brief explanations of these quantities.

The symbols are briefly explained in Table 1, and some of them are illustrated in Figure 1. Pedigree members with unspecified mother and father are called the founders of the pedigree, and the other members are non-founders. The recursive algorithm presupposes that pedigree members are ordered under the constraint that parents always precede children. We denote by subscripts *a*, *b*, *c* the members of a pedigree, and denote by a>b individual *a* comes after individual *b* (≠a). We denote by superscripts *m* the maternally derived genes or chromosomes, and *p* for the paternally derived. We denote by superscripts *o*, $o_{1}$, $o_{2}$, and $o_{3}$ the unspecified parental origins (*m* or *p*) of genes or chromosomes. For the sake of brevity, f(a)={AA,XX,XY} denotes a horizontal piece-wise equation, so that f(a)=AA, XX, and XY for the autosomes of individual *a*, XX chromosomes of female *a*, and XY chromosomes of male *a*, respectively.

**Table 1:** List of symbols and their brief descriptions

Symbol	Description
$1_{S}$	An indicator function that equals 1 if statement *S* is true and 0 otherwise
$0_{S1}^{S2}$	An indicator function that equals 0 if S1 and S2 are true and 1 otherwise.
$o,o_{1},o_{2},o_{3}$	A gene or haplotype is maternally (=m) or paternally (=p) derived
a,b,c	Pedigree members
♂, ♀	Father of *a*, mother of *a*
$\varphi_{ab}^{mp}\left(11\right)$	Probability of IBD between maternal gene of *a* and paternal gene of *b*
$\varphi_{ab}^{o_{1}o_{2}}\left(11\right)$	Probability of IBD between two genes of ab with parental origins $o_{1}o_{2}$
$\varphi_{ab}^{o_{1}o_{2}}\left(12\right)$	Probability of non-IBD between two genes of ab with parental origins $o_{1}o_{2}$
$\varphi_{abc}^{o_{1}o_{2}o_{3}}\left(123\right)$	Probability of non-IBD among three genes of abc with parental origins $o_{1}o_{2}o_{3}$
$R_{a}^{m}$	Expected junction density (per Morgan) on the maternal chromosome of *a*
$R_{a}^{o}$	On the chromosome of *a* with parental origin *o*
$\left(g_{1}g_{2}g_{3}g_{4}\right)$	A junction type denoted by two-locus four-gene identity state: $g_{1}g_{2}$ ($g_{3}g_{4}$) are
	on the left-hand (right-hand) side of junction, and $g_{1}g_{3}$ and $g_{2}g_{4}$ are two haplotypes.
	Seven types: 1112, 1121, 1122, 1211, 1213, 1222, and 1232
$J_{ab}^{o_{1}o_{2}}\left(g_{1}g_{2}g_{3}g_{4}\right)$	Expected density of junction type $\left(g_{1}g_{2}g_{3}g_{4}\right),$ where haplotype $g_{1}g_{3}$ of *a* has
	parental origin $o_{1}$ and haplotype $g_{2}g_{4}$ of *b* has parental origin $o_{2}$
$\rho_{aa}^{mp}$	Expected overall junction density for *a*. $\rho_{aa}^{mp}=R_{a}^{m}+R_{a}^{p}-J_{aa}^{mp}\left(1122\right)$
Ω	A set of distinct FGLs assigned to the founders of a pedigree, i,j,k∈Ω
i≠j≠k	FGLs i,j,k differ from each other so that i≠j, j≠k, and i≠k
$\varphi_{a}^{m}\left(i\right)$	Probability that the maternal gene of *a* has FGL *i*
$\varphi_{a}^{o}\left(i\right)$	Probability that the gene of *a* with parental origin *o* has FGL *i*. $\sum_{i\in \text{Ω}}\varphi_{a}^{o}\left(i\right)=1$
$\varphi_{ab}^{mp}\left(ij\right)$	Probability that the maternal gene of *a* has FGL *i* and the paternal gene of *b* has FGL *j*.
$\varphi_{ab}^{o_{1}o_{2}}\left(ij\right)$	Probability that two genes of ab with parental origins $o_{1}o_{2}$ have FGLs ij
	$\varphi_{ab}^{o_{1}o_{2}}\left(12\right)=\sum_{i,j\in \text{Ω},i\neq j}\varphi_{ab}^{o_{1}o_{2}}\left(ij\right)$
$\varphi_{abc}^{o_{1}o_{2}o_{3}}\left(ijk\right)$	Probability that three genes of abc with parental origins $o_{1}o_{2}o_{3}$ have FGLs ijk.
	$\varphi_{abc}^{o_{1}o_{2}o_{3}}\left(123\right)=\sum_{i,j,k\in \text{Ω},i\neq j\neq k}\varphi_{abc}^{o_{1}o_{2}o_{3}}\left(ijk\right)$
$R_{a}^{o}\left(ij\right)$	Expected junction density (per Morgan) on chromosome of *a* with parental origin *o*,
	where the left-hand (right-hand) side of junction has FGL *i*(j≠i). $R_{a}^{o}=\sum_{i,j\in \text{Ω},i\neq j}R_{a}^{o}\left(ij\right)$
$J_{ab}^{o_{1}o_{2}}\left(ijkj\right)$	Expected junction density: the four genes have FGLs ijkj, haplotype ik of *a* has parental origin $o_{1}$,
	and haplotype jj of *b* has parental origin $o_{2}$. $J_{ab}^{o_{1}o_{2}}\left(1232\right)=\sum_{i,j,k\in \Omega ,i\neq j\neq k}J_{ab}^{o_{1}o_{2}}\left(ijkj\right)$.
	Similarly for $J_{ab}^{o_{1}o_{2}}\left(iiij\right),$ $J_{ab}^{o_{1}o_{2}}\left(iiji\right),$ $J_{ab}^{o_{1}o_{2}}\left(iijj\right),$ $J_{ab}^{o_{1}o_{2}}\left(ijii\right),$ $J_{ab}^{o_{1}o_{2}}\left(ijik\right),$ $J_{ab}^{o_{1}o_{2}}\left(ijjj\right)$

**Figure 2:**
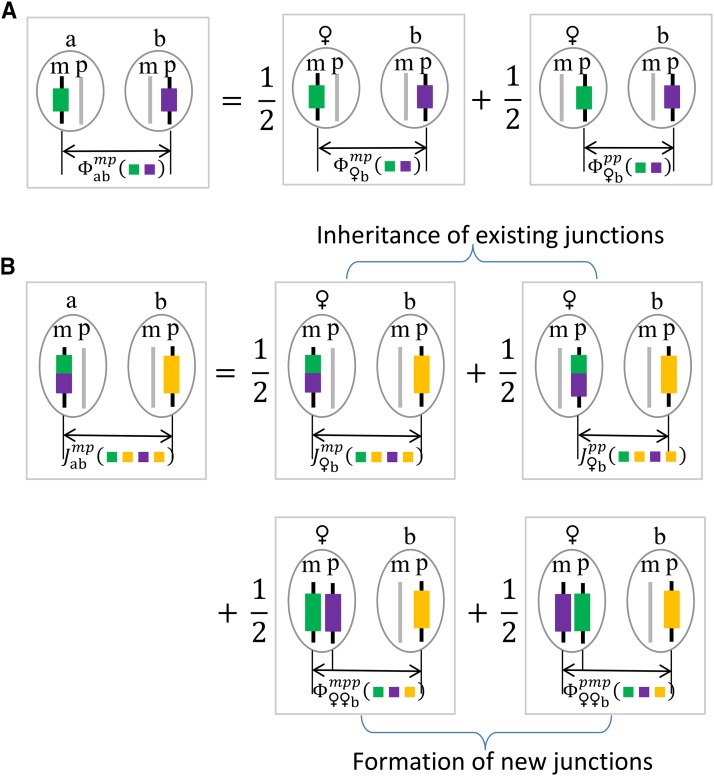
Pictorial representation of recurrence equations. (A) The equation for ancestral coefficient $\varphi_{ab}^{mp}\left(ij\right)$ (a>b,). (B) The equation for ancestral junction density $J_{ab}^{mp}\left(ijkj\right)$ (a>b). The FGLs *i*, *j*, and *k* are shown by different colors, and the irrelevant chromosomes are shown as gray. The symbol ♀ denotes the mother of individual *a*, and see Table 1 for other notations.

In the derivations of recurrence relations of a quantity, we will focus on a particular ordering a>b if the quantity concerns genes in two individuals; we will always trace the maternally derived gene or chromosome within non-founder *a* back to its two parental genes or chromosomes if the maternally derived gene or chromosome concerns the quantity, and otherwise trace the paternally derived gene or chromosome. Throughout this paper, ♂ and ♀ always denote the father and mother of individual *a*, rather than any other, respectively.

The identity coefficient $\varphi_{ab}^{o_{1}o_{2}}\left(12\right)$ denotes that two genes at a single locus have identity state (12), where the first gene is in individual *a* and has parental origin $o_{1},$ and the second gene is in individual *b* and has parental origin $o_{2}$ (Figure 1A). There are only two two-gene identity states: IBD (11) or non-IBD (12), and $\varphi_{ab}^{o_{1}o_{2}}\left(11\right)=1-\varphi_{ab}^{o_{1}o_{2}}\left(12\right).$ Similarly, we denote by $\varphi_{abc}^{o_{1}o_{2}o_{3}}\left(123\right)$ the three-gene identity coefficient with the identity state being the non-IBD state (123), which is required together with two-gene identity coefficients for deriving the recursive relations for junction densities.

An expected identity junction density is defined as the expected number of the specified type of recombination breakpoints per Morgan along two homologous chromosomes. Here expectation concerns the stochasticity of gene flow from founders to descendants on a fixed pedigree. And identity indicates that only the identity patterns (not specific FGLs) on the two sides of junctions matter. In the recursive algorithm NON-EXCH, we will introduce ancestral junction densities where the specific FGLs do matter. For example, $J_{ab}^{o_{1}o_{2}}\left(1232\right)$ denotes that the four genes, two at each side of a breakpoint along two chromosomes, have genetic identity state (1232) (Figure 1B). Here the first and third genes are on the left and right sides of a breakpoint, respectively, and they are in individual *a* and have parental origin $o_{1}.$ And the second and fourth genes are on the left and right sides, respectively, and they are in individual *b* and have parental origin $o_{2}.$ We adopt the notation of Nadot and Vayssiex (1973) for an identity state: the genes are labeled by natural integers starting 1, and the same integer is assigned to the gene that is IBD with a previous gene.

Following the previous framework (Zheng *et al.* 2014; Zheng 2015), we model ancestral origins along two homologous chromosomes within an individual by a continuous time Markov process, which can be described by an initial distribution *π* of ancestral origins and a rate matrix *Q*. Under the assumption of exchangeability among FGLs, the initial distribution *π* of individual *a* is determined by the identity coefficient $\varphi_{aa}^{mp}\left(11\right),$ and the rate matrix *Q* of individual *a* is determined by the five expected identity junction densities $J_{aa}^{mp}\left(1122\right),$ $J_{aa}^{mp}\left(1211\right),$ $J_{aa}^{mp}\left(1213\right),$ $J_{aa}^{mp}\left(1222\right),$ and $J_{aa}^{mp}\left(1232\right);$ see Zheng (2015) for an illustrative construction of rate matrix *Q* from the five junction densities.

In the following, we describe a recursive algorithm for calculating the identity coefficients and the expected junction densities for an individual in a given pedigree. In short, let $1_{S}$ be an indicator function that equals 1 if statement *S* is true and 0 otherwise, and let $0_{S1}^{S2}=1-1_{S1}1_{S2}$ be an indicator function that equals 0 if statements S1 and S2 are true and 1 otherwise.

### Identity coefficients

The recurrence relations for the two- and three-gene identity coefficients are similar to the recursive algorithm of Karigl (1981). However, the calculation proceeds by tracing back genes instead of individuals, see also *e.g.*, Jacquard (1974), Nadot and Vayssiex (1973), Denniston (1974), and Garcia-Cortes (2015). Thus, we can account for the asymmetry between maternally and paternally derived chromosomes, and account for autosomes and sex chromosomes simultaneously. Throughout this paper, we assume that there are no crossovers between X and Y sex chromosomes within a male. In addition, we focus on non-IBD state (12) instead of IBD state (11) to simplify the recurrence relations.

We derive the recurrence relations according to the Mendelian inheritance rules: (1) a maternally (or paternally) derived autosomal gene descends from the two genes within the mother (or father, respectively) with equal probability 1/2, (2) similar to (1) for a maternally derived X-linked gene, and (3) a paternally derived sex-linked gene within a female (or male) must descend from the X-linked (or Y-linked, respectively) gene of the father. The recurrence relations of the two-gene identity coefficient $\varphi_{ab}^{o_{1}o_{2}}\left(12\right)$ for non-founder *a* are given by

$$\varphi_{ab}^{mo}\left(12\right)=\frac{1}{2}\left[\varphi_{♀b}^{mo}\left(12\right)+\varphi_{♀b}^{po}\left(12\right)\right]0_{a=b}^{m=o},a\geq b\varphi_{ab}^{po}\left(12\right)=\left\{\frac{1}{2}\left[\varphi_{♂b}^{mo}\left(12\right)+\varphi_{♂b}^{po}\left(12\right)\right],\varphi_{♂b}^{mo}\left(12\right),\varphi_{♂b}^{po}\left(12\right)\right\}0_{a=b}^{p=o},a\geq b,\varphi_{ab}^{o_{1}o_{2}}\left(12\right)=\varphi_{ba}^{o_{2}o_{1}}\left(12\right),a<b,$$

for $o,o_{1},o_{2}\in \left\{m,p\right\}.$ Here the first equation follows from the rules 1 and 2 for the maternally derived gene within individual *a*, the second equation follows from the rules 1 and 3 for the paternally derived gene within individual *a*, and the last equation is obtained by reversing the ordering of two genes. The symmetries such as $\varphi_{abc}^{o_{1}o_{2}o_{3}}\left(123\right)=\varphi_{acb}^{o_{1}o_{3}o_{2}}\left(123\right)=\ldots =\varphi_{cba}^{o_{3}o_{2}o_{1}}\left(123\right)$ apply to three-gene identity coefficients by permuting the three genes, and thus we consider only a particular ordering a≥b≥c, and we have

$$\varphi_{abc}^{mo_{1}o_{2}}\left(123\right)=\frac{1}{2}\left[\varphi_{♀bc}^{mo_{1}o_{2}}\left(123\right)+\varphi_{♀bc}^{po_{1}o_{2}}\left(123\right)\right]0_{a=b}^{m=o_{1}}0_{a=c}^{m=o_{2}}0_{b=c}^{o_{1}=o_{2}},\varphi_{abc}^{po_{1}o_{2}}\left(123\right)=\left\{\frac{1}{2}\left[\varphi_{♂bc}^{mo_{1}o_{2}}\left(123\right)+\varphi_{♂bc}^{po_{1}o_{2}}\left(123\right)\right],\varphi_{♂bc}^{mo_{1}o_{2}}\left(123\right),\varphi_{♂bc}^{po_{1}o_{2}}\left(123\right)\right\}0_{a=b}^{p=o_{1}}0_{a=c}^{p=o_{2}}0_{b=c}^{o_{1}=o_{2}},$$

for $o_{1},o_{2}\in \left\{m,p\right\},$ where the first (second) equation traces the maternally (or paternally, respectively) derived gene within individual *a*.

The boundary conditions of these recurrence relations are given according to the assignment of FGLs to the founders in a pedigree. If we assign distinct FGLs to each haploid genome of an outbred founder, all multi-gene non-IBD probabilities are 1 if any pair of genes is not from the same haploid genome, and 0 otherwise. Four-gene or higher order identity coefficients may be derived similarly, but they are not required for the derivations of junction densities.

### Expected identity junction densities

Let $R_{a}^{m}$ $\left(R_{a}^{p}\right)$ be the maternal (paternal) map expansion, the expected density of recombination breakpoints on the maternally (paternally) derived chromosome of individual *a*. The implicit two-gene identity state for the map expansions is (12). It holds that

$$\begin{array}{c} R_{a}^{m}=J_{aa}^{mp}\left(1122\right)+J_{aa}^{mp}\left(1121\right)+J_{aa}^{mp}\left(1222\right)+J_{aa}^{mp}\left(1232\right),\\ R_{a}^{p}=J_{aa}^{mp}\left(1122\right)+J_{aa}^{mp}\left(1112\right)+J_{aa}^{mp}\left(1211\right)+J_{aa}^{mp}\left(1213\right), \end{array}$$

where $J_{aa}^{mp}\left(1121\right)=J_{aa}^{mp}\left(1222\right)$ and $J_{aa}^{mp}\left(1112\right)=J_{aa}^{mp}\left(1211\right)$ according to the reversibility of chromosome direction (Zheng 2015). The expected overall junction density for individual *a* is given by $\rho_{aa}^{mp}=R_{a}^{m}+R_{a}^{p}-J_{aa}^{mp}\left(1122\right).$ In the following, we derive the recurrence relations for the five expected junction densities $R_{a}^{m},$ $R_{a}^{p},$ $J_{aa}^{mp}\left(1122\right),$ $J_{aa}^{mp}\left(1213\right),$ and $J_{aa}^{mp}\left(1232\right),$ from which $J_{aa}^{mp}\left(1211\right)$ and $J_{aa}^{mp}\left(1222\right)$ can be derived according to the above equations of map expansions. The recurrence relations for the two map expansions of non-founder *a* are relatively simple, and they are given by (Zheng 2015)

$$\begin{array}{c} R_{a}^{m}=\frac{1}{2}\left(R_{♀}^{m}+R_{♀}^{p}\right)+\varphi_{\mathrm{♀♀}}^{mp}\left(12\right),\\ R_{a}^{p}=\left\{\frac{1}{2}\left(R_{♂}^{m}+R_{♂}^{p}\right)+\varphi_{\mathrm{♂♂}}^{mp}\left(12\right),R_{♂}^{m},R_{♂}^{p}\right\}, \end{array}$$

according to the theory of junctions that a new identity junction is formed whenever a recombination event occurs between two homologous chromosomes that are non-IBD at the location of a crossover (Fisher 1954).

Since chromosomes are modeled as a genomic continuum, the shared junction type (1122) between two chromosomes can be formed only by duplicating the chromosome segment harboring the recombination breakpoint. For the expected junction density $J_{aa}^{o_{1}o_{2}}\left(1122\right)$ of non-founder *a*, we have

$$J_{aa}^{oo}\left(1122\right)=R_{a}^{o},J_{aa}^{mp}\left(1122\right)=J_{aa}^{pm}\left(1122\right)=\frac{1}{2}\left[J_{a♀}^{pm}\left(1122\right)+J_{a♀}^{pp}\left(1122\right)\right],J_{ab}^{mo}\left(1122\right)=\frac{1}{2}\left[J_{♀b}^{mo}\left(1122\right)+J_{♀b}^{po}\left(1122\right)\right],a>b,J_{ab}^{po}\left(1122\right)=\left\{\frac{1}{2}\left[J_{♂b}^{mo}\left(1122\right)+J_{♂b}^{po}\left(1122\right)\right],J_{♂b}^{mo}\left(1122\right),J_{♂b}^{po}\left(1122\right)\right\},a>b,J_{ab}^{o_{1}o_{2}}\left(1122\right)=J_{ba}^{o_{2}o_{1}}\left(1122\right),a<b,$$

for $o,o_{1},o_{2}\in \left\{m,p\right\},$ where and the last equation is obtained by reversing the ordering of the two haplotypes. In the first equation, $J_{aa}^{oo}\left(1122\right)$ refers to the same chromosome in individual *a*, and thus equals $R_{a}^{o}$ by definition. We trace the maternally derived haplotype within non-founder *a* back to its two parental haplotypes if the maternally derived haplotype concerns the junction density, and otherwise track the paternally derived haplotype.

We derive the recurrence relations for $J_{ab}^{o_{1}o_{2}}\left(1213\right)$ and $J_{ab}^{o_{1}o_{2}}\left(1232\right)$ jointly. The recombination breakpoint for (1232) occurs on the first chromosome of a homologous pair, and it is on the second chromosome for (1213). The junction type (1213) becomes (1232) when reversing the ordering of two haplotype of junction type (1213). We have

$$J_{ab}^{mo}\left(1232\right)=\frac{1}{2}\left[J_{♀b}^{mo}\left(1232\right)+J_{♀b}^{po}\left(1232\right)\right]0_{a=b}^{m=o}+\varphi_{\mathrm{♀♀}b}^{mpo}\left(123\right)0_{a=b}^{m=o},a\geq b,J_{ab}^{po}\left(1232\right)=\left\{\frac{1}{2}\left[J_{♂b}^{mo}\left(1232\right)+J_{♂b}^{po}\left(1232\right)\right]+\varphi_{\mathrm{♂♂}b}^{mpo}\left(123\right),J_{♂b}^{mo}\left(1232\right),J_{♂b}^{po}\left(1232\right)\right\}0_{a=b}^{p=o},a\geq b,J_{ab}^{o_{1}o_{2}}\left(1232\right)=J_{ba}^{o_{2}o_{1}}\left(1213\right),a<b,J_{ab}^{mo}\left(1213\right)=\frac{1}{2}\left[J_{♀b}^{mo}\left(1213\right)+J_{♀b}^{po}\left(1213\right)\right]0_{a=b}^{m=o},a\geq b,J_{ab}^{po}\left(1213\right)=\left\{\frac{1}{2}\left[J_{♂b}^{mo}\left(1213\right)+J_{♂b}^{po}\left(1213\right)\right],J_{♂b}^{mo}\left(1213\right),J_{♂b}^{po}\left(1213\right)\right\}0_{a=b}^{p=o},a\geq b,J_{ab}^{o_{1}o_{2}}\left(1213\right)=J_{ba}^{o_{2}o_{1}}\left(1232\right),a<b,$$

for $o,o_{1},o_{2}\in \left\{m,p\right\}.$ There are no contributions from the three-gene non-IBD probabilities in the equations for $J_{ab}^{mo}\left(1213\right)$ and $J_{ab}^{po}\left(1213\right),$ because crossovers between the two parental haplotypes of the tracing haplotype within individual *a* are invisible.

The boundary conditions are given by the assignment of FGLs to the founders. The within- and between-founder identity junction densities are 0 if a single FGL is assigned to the whole haploid genome of each founder.

## Recursive Algorithm Non-Exch

### Notations and overview

Let Ω denote the set of distinct FGLs assigned to the founders of a pedigree. We adopt the same notations in the recursive algorithm with FGL exchangeability except for the following changes. As shown in Figure 1, we replace identity states by ancestral states: (1122)→(iijj), (1211)→(ijii), (1213)→(ijik), (1222)→(ijjj), and (1232)→(ijkj) for the two-chromosome expected junction densities, and $R_{a}^{m}\to R_{a}^{m}\left(ij\right)$ and $R_{a}^{p}\to R_{a}^{p}\left(ij\right)$ explicitly for the map expansions, where i,j,k∈Ω are different from each other (denoted by i≠j≠k).

We represent a two-gene ancestral state by (ij), and a three-gene ancestral state by (ijk), where i,j,k∈Ω are not necessary different from each other. In addition we introduce one-gene ancestral coefficient $\varphi_{a}^{o}\left(i\right),$ the probability that the maternally (o=m) or paternally (o=p) derived gene in individual *a* carries FGL i∈Ω. The identity coefficients can be obtained by summing the corresponding ancestral coefficients. For example, $\varphi_{ab}^{o_{1}o_{2}}\left(11\right)=\sum_{i\in \text{Ω}}\varphi_{ab}^{o_{1}o_{2}}\left(ii\right).$ Similar relationships hold between the expected identity junction densities and the expected ancestral junction densities, for example, $J_{ab}^{o_{1}o_{2}}\left(1122\right)=\sum_{i,j\in \text{Ω},i\neq j}J_{ab}^{o_{1}o_{2}}\left(iijj\right)$ and $R_{a}^{o}=\sum_{i,j\in \text{Ω},i\neq j}R_{a}^{o}\left(ij\right).$

Similarly, the initial distribution *π* of the Markov chain can be constructed from the two-gene ancestral coefficients, and the rate matrix *Q* can be constructed from the expected ancestral junction densities, without assuming the exchangeability of FGLs.

### Ancestral coefficients

Bauman *et al.* (2008) and Zhou *et al.* (2012) derived the recurrence relations of the one- and two-gene ancestral coefficients for autosomes by tracing two distinct genes simultaneously in their parents. We derive equivalent recurrence relations for both autosomes and sex chromosomes by tracing one gene once in its parent, instead of simultaneously tracing two genes within an individual. In addition, we derive the recurrent relations of the three-gene ancestral coefficients that are required in the recurrence relations of the expected ancestral junction densities.

The relations of the ancestral coefficients are very similar to those of the identity coefficients, and they are based on the same rules of Mendelian inheritance. For example for the one-gene ancestral coefficient $\varphi_{a}^{o}\left(i\right)$ of non-founder *a*, we have

$$\begin{array}{c} \varphi_{a}^{m}\left(i\right)=\frac{1}{2}\left[\varphi_{♀}^{m}\left(i\right)+\varphi_{♀}^{p}\left(i\right)\right],\\ \varphi_{a}^{p}\left(i\right)=\left\{\frac{1}{2}\left[\varphi_{♂}^{m}\left(i\right)+\varphi_{♂}^{p}\left(i\right)\right],\varphi_{♂}^{m}\left(i\right),\varphi_{♂}^{p}\left(i\right)\right\}. \end{array}$$

See Appendix A for the recurrence relations of the two-gene ancestral coefficient $\varphi_{abc}^{o_{1}o_{2}}\left(ij\right)$ and three-gene ancestral coefficient $\varphi_{abc}^{o_{1}o_{2}o_{3}}\left(ijk\right).$

The boundary conditions of the recurrence relations of the ancestral coefficients are given by the FGLs assigned to the founders in a pedigree.

### Expected ancestral junction densities

The ancestral map expansions can be expressed in terms of the expected ancestral junction densities as follows

$$\begin{array}{l} R_{a}^{m}\left(ij\right)=J_{aa}^{mp}\left(iijj\right)+J_{aa}^{mp}\left(iiji\right)+J_{aa}^{mp}\left(ijjj\right)+\underset{k\in \text{Ω},i\neq j\neq k}{\sum }J_{aa}^{mp}\left(ikjk\right),\\ R_{a}^{p}\left(ij\right)=J_{aa}^{mp}\left(iijj\right)+J_{aa}^{mp}\left(iiij\right)+J_{aa}^{mp}\left(jijj\right)+\underset{k\in \text{Ω},i\neq j\neq k}{\sum }J_{aa}^{mp}\left(kikj\right). \end{array}$$

Here $R_{a}^{o}\left(ij\right),o\in \left\{m,p\right\}$ refers to the junctions on the single chromosome of *a* with parental origin *o*, while the terms on the right-hand side refer to the junctions on the two homologous chromosomes of *a*. We have $J_{aa}^{mp}\left(iiji\right)=J_{aa}^{mp}\left(jiii\right)\neq J_{aa}^{mp}\left(ijjj\right)$ and $J_{aa}^{mp}\left(iiij\right)=J_{aa}^{mp}\left(ijii\right)\neq J_{aa}^{mp}\left(jijj\right),$ where the equal signs are based on the reversibility of chromosome direction, and the unequal signs refer to the non-exchangeability of FGLs *i* and *j*.

The recurrence relations for the ancestral map expansions are given by

$$R_{a}^{m}\left(ij\right)=\frac{1}{2}\left[R_{♀}^{m}\left(ij\right)+R_{♀}^{p}\left(ij\right)\right]+\frac{1}{2}\left[\varphi_{\mathrm{♀♀}}^{mp}\left(ij\right)+\varphi_{\mathrm{♀♀}}^{pm}\left(ij\right)\right]1_{i\neq j},R_{a}^{p}\left(ij\right)=\left\{\frac{1}{2}\left[R_{♂}^{m}\left(ij\right)+R_{♂}^{p}\left(ij\right)\right]+\frac{1}{2}\left[\varphi_{\mathrm{♂♂}}^{mp}\left(ij\right)+\varphi_{\mathrm{♂♂}}^{pm}\left(ij\right)\right]1_{i\neq j},R_{♂}^{m}\left(ij\right),R_{♂}^{p}\left(ij\right)\right\},$$

where the contributions of the two-gene ancestral coefficients account for the asymmetry of FGLs *i* and *j*, for example, $\varphi_{\mathrm{♀♀}}^{mp}\left(ij\right)=\varphi_{\mathrm{♀♀}}^{pm}\left(ji\right)\neq \varphi_{\mathrm{♀♀}}^{pm}\left(ij\right).$ A new ancestral junction is formed whenever a recombination event occurs between two homologous chromosomes that have the unordered FGLs *i* and *j* at the location of a crossover.

The recurrence relations for $J_{ab}^{o_{1}o_{2}}\left(iijj\right)$ are the same as those for $J_{ab}^{o_{1}o_{2}}\left(1122\right),$ except that identity states are replaced by ancestral states. We have

$$\begin{array}{l} J_{aa}^{oo}\left(iijj\right)=R_{a}^{o}\left(ij\right),\\ J_{aa}^{mp}\left(iijj\right)=J_{aa}^{pm}\left(iijj\right)=\frac{1}{2}\left[J_{a♀}^{pm}\left(iijj\right)+J_{a♀}^{pp}\left(iijj\right)\right],\\ J_{ab}^{mo}\left(iijj\right)=\frac{1}{2}\left[J_{♀b}^{mo}\left(iijj\right)+J_{♀b}^{po}\left(iijj\right)\right],a>b,\\ J_{ab}^{po}\left(iijj\right)=\left\{\frac{1}{2}\left[J_{♂b}^{mo}\left(iijj\right)+J_{♂b}^{po}\left(iijj\right)\right],J_{♂b}^{mo}\left(iijj\right),J_{♂b}^{po}\left(iijj\right)\right\},a>b,\\ J_{ab}^{o_{1}o_{2}}\left(iijj\right)=J_{ba}^{o_{2}o_{1}}\left(jjii\right),a<b, \end{array}$$

for $o,o_{1},o_{2}\in \left\{m,p\right\},$ where the last equation is obtained by reversing the ordering of the two haplotypes. See Appendix B for the recurrence relations of $J_{ab}^{o_{1}o_{2}}\left(ijkj\right),$ $J_{ab}^{o_{1}o_{2}}\left(ijik\right)$ $J_{ab}^{o_{1}o_{2}}\left(ijjj\right)$, and $J_{ab}^{o_{1}o_{2}}\left(ijii\right).$

As before, the within- and between-founder ancestral junction densities are 0 if a single FGL is assigned to the whole haploid genome of each founder.

### Data availability

Ancestral inference using the two recursive algorithms is implemented in the previous developed RABBIT software, where the function magicReconstruct is extended to incorporate pedigree information. RABBIT is available at https://github.com/chaozhi/RABBIT.git, and it is offered under the GNU Affero general public license, version 3 (AGPL-3.0). Example scripts for using the recursive algorithms, simulating genotypic data, and ancestral inference are included. The real CC data have been described by Durrant *et al.* (2011).

## Application to Multiparental Populations

### Simulation evaluation

**Figure 3:**
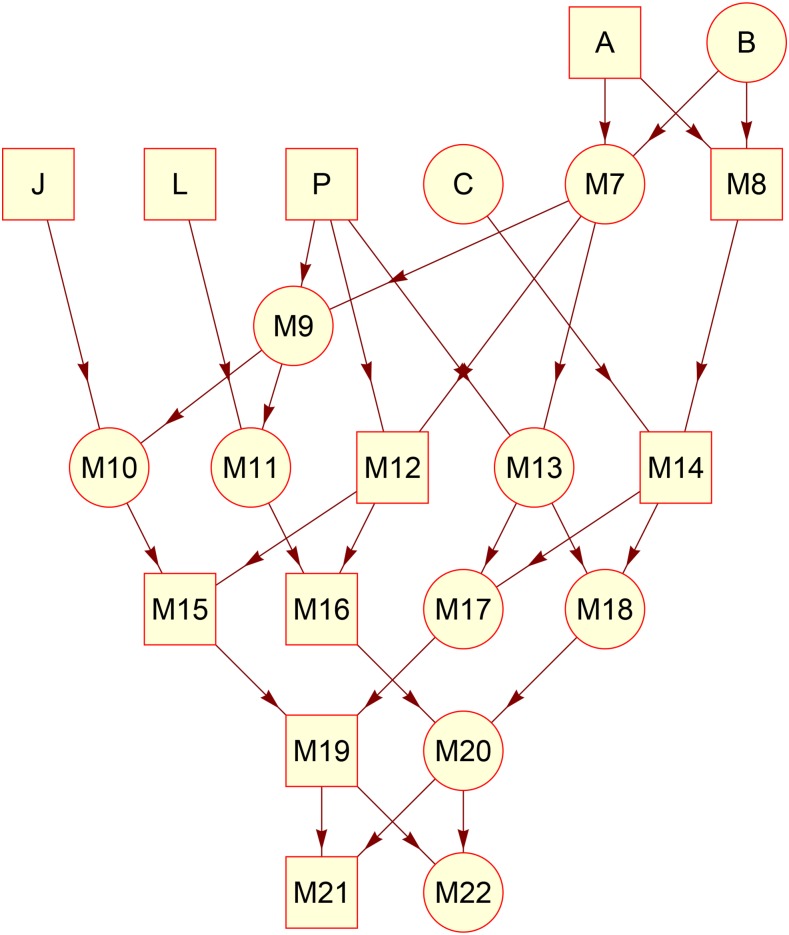
The pedigree of Native Americans. It consists of 6 founders and 16 non-founders. Circles denote females, and rectangles for males.

Before applying the two recursive algorithms EXCH and NON-EXCH to multiparental populations, we evaluate their accuracy by forward simulations on the classical pedigree of Native Americans (Figure 3) (Chapman and Jacquard 1971). The pedigree has been previously used for evaluating recursive algorithms (Jacquard 1974; Karigl 1981; Garcia-Cortes 2015). We simulate two linkage groups: one pair of homologous autosomes and one pair of sex chromosomes. We assign FGLs to the haploid or diploid genomes of the founders. Each descendant gamete is specified as a list of FGL segments determined by chromosomal crossovers. The number of crossovers in a linkage group of a gamete follows a Poisson distribution with mean 1. We assume no genetic interference so that the positions of crossovers are independently and randomly distributed across the chromosomes. We obtain simulated results for the pedigree member “M22” (Figure 3) by averaging over $10^{6}$ simulations.

Table 2 shows the comparisons between the numerical results from the recursive algorithm EXCH and the simulated results. A unique FGL is assigned to each haploid genome of each founder, so that in total twelve distinct FGLs are assigned to the fixed founders. The differences between the numerical and simulated results are less than 0.002, which is very likely due to the stochasticity of gene flow from founders to descendants. The identity coefficient $\varphi_{aa}^{mp}\left(11\right)$ for autosomes is in agreement with the previous result (Karigl 1981).

**Table 2:** The identity coefficients and the expected identity junction densities for pedigree member ”M22”

Quantity *^a^*	AA autosomes		XX autosomes	
	Numerical	Simulated	Numerical	Simulated
$\varphi_{aa}^{mp}\left(11\right)$	0.18359	0.18324	0.23437	0.23459
$J_{aa}^{mp}\left(1122\right)$	0.18359	0.18371	0.17969	0.17969
$J_{aa}^{mp}\left(1211\right)$	0.61572	0.61516	0.35938	0.35978
$J_{aa}^{mp}\left(1213\right)$	2.93652	2.93462	1.47656	1.47587
$J_{aa}^{mp}\left(1222\right)$	0.61572	0.61605	0.57031	0.57047
$J_{aa}^{mp}\left(1232\right)$	2.93652	2.93824	1.71094	1.71227

a A unique FGL is assigned to each haploid genome of each founder in Figure 3.

Table 3 evaluates the recursive algorithm NON-EXCH by the forward simulations. A unique FGL is assigned to the whole diploid genome of each founder, so that in total six distinct FGLs are assigned to the fixed founders. Here we use a different assignment of FGLs because Table 3 was otherwise too large. The results show that founder “J” does not contribute to the maternally derived autosome and X chromosome in offspring “M22”, which is relatively straightforward from the pedigree structure in Figure 3. The differences between the numerical and simulated results, including those for $\varphi_{aa}^{mp}\left(ij\right),$ $J_{aa}^{mp}\left(iijj\right),$ $J_{aa}^{mp}\left(ijii\right),$ $J_{aa}^{mp}\left(ijik\right),$ $J_{aa}^{mp}\left(ijjj\right),$ and $J_{aa}^{mp}\left(ijkj\right),$ are less than 0.001.

**Table 3:** The ancestral coefficients and the expected ancestral junction densities for pedigree member ”M22”

Quantity *^a^*	AA autosomes		XX autosomes	
	Numerical	Simulated	Numerical	Simulated
$\varphi_{a}^{m}\left(i\right)$				
A	0.21875	0.21885	0.125	0.12507
B	0.21875	0.21874	0.125	0.12467
J	0	0	0	0
L	0.125	0.12487	0.25	0.25078
P	0.3125	0.31241	0.25	0.24939
C	0.125	0.12513	0.25	0.25009
$R_{a}^{m}\left(ij\right)$				
AB	0.31250	0.31272	0.14062	0.14098
AJ	0	0	0	0
AL	0.09375	0.09348	0.09375	0.09364
AP	0.28906	0.28880	0.15625	0.15602
AC	0.11719	0.11768	0.09375	0.09330
BJ	0	0	0	0
BL	0.09375	0.09405	0.09375	0.09384
BP	0.28906	0.28890	0.15625	0.15626
BC	0.11719	0.11742	0.09375	0.09331
JL	0	0	0	0
JP	0	0	0	0
JC	0	0	0	0
LP	0.15625	0.15644	0.18750	0.18748
LC	0.03125	0.03124	0.12500	0.12422
PC	0.10938	0.10922	0.18750	0.18735

a The label of each founder is used as FGL that is assigned to its diploid genomes.

**Figure 4:**
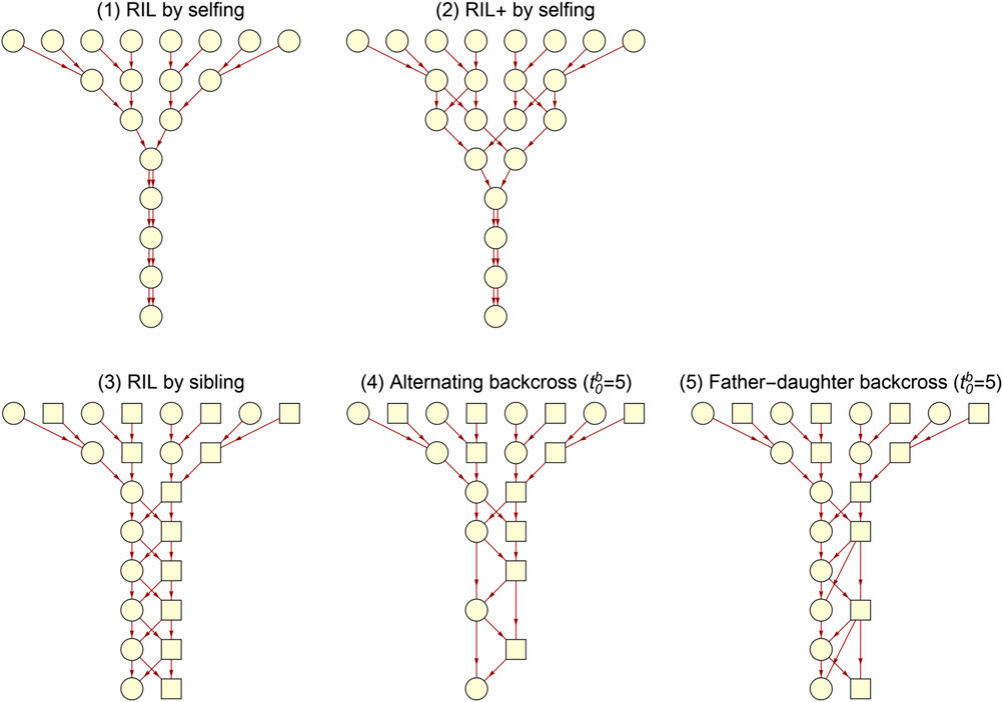
Illustration of different 8-way funnel breeding schemes. The alternating backcross scheme alternates between mother-son and father-daughter matings in generation $t\geq t_{0}^{b},$ and the father-daughter backcross alternates between father-daughter and random sibling matings in generation $t\geq t_{0}^{b}.$ Circles denote females, and rectangles for males.

We confirmed that the numerical results from the algorithm NON-EXCH are reduced to those from the algorithm EXCH, by summing over possible FGLs under the given identity pattern. In addition, we confirmed the consistency between the two algorithms in the multiparental populations shown in Figure 4.

### Breeding design

We apply the recursive algorithm EXCH to multiparental populations, which have become attractive for QTL mapping in many animal and plant species. Specifically, we study the inbreeding process by calculating the IBD probability $\varphi_{aa}^{mp}\left(11\right),$ and measure the QTL mapping resolution by calculating the expected overall junction density $\rho_{aa}^{mp}.$ We focus on multi-way funnel breeding schemes (Figure 4). In the funnel scheme, the founders of each line are randomly permuted, and each line is produced by a intercross scheme that combines all founder genomes through several generations of pairwise crosses prior to repeated generations of inbreeding by *e.g.*, sibling mating. The alternating backcross and the father-daughter backcross have been studied by Welsh and McMillan (2012) for accelerating inbreeding by a simulation approach.

**Figure 5:**
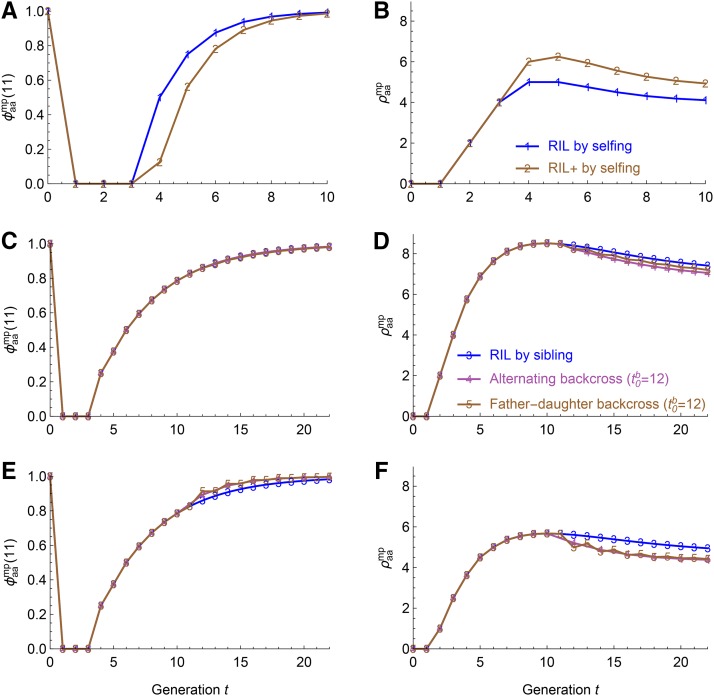
The identity coefficient $\varphi_{aa}^{mp}\left(11\right)$ and the expected overall junction density $\varphi_{aa}^{mp}$ for the breeding schemes in Figure 4. (A-B) The results for selfing mating schemes 1-2 in Figure 4. (C-D) The results for the autosomes of mating schemes 3-5 with backcross starting from $t_{0}^{b}=12$ in Figure 4. (E-F) The results for the sex chromosomes.

Figure 5A and B show the IBD probability and the expected overall junction density as a function of generation for the recombinant inbred lines (RIL) by selfing and the RIL+ by selfing. These plant breeding schemes have been adopted in many recently produced crop multiparent advanced generation intercross (MAGIC) populations (*e.g.*, Huang *et al.* 2012; Mackay *et al.* 2014; Pascual *et al.* 2015). The RIL+ by selfing has one additional generation of intercrossing instead of selfing in the RIL scheme (Teuscher and Broman 2007). Thus, the RIL+ scheme has smaller IBD probability $\varphi_{aa}^{mp}\left(11\right)$ (Figure 5A), and has about 1 per M larger overall junction density $\rho_{aa}^{mp}$ in a given later generation (t≥5)(Figure 5B).

Figure 5C and E show that the IBD probabilities $\varphi_{aa}^{mp}\left(11\right)$ for the alternating backcross and the father-daughter backcross are the same as those of the RIL by sibling for autosomes and they are smaller than those of the RIL by sibling for sex chromosomes. Welsh and McMillan (2012) measured the inbreeding process by the number of generations to reach complete fixation of genome as homozygous, and showed that the number of generation increases from the alternating backcross to the father-daughter backcross and to the RIL by sibling. The differences in the number of generation can be partially because the differences of the IBD probabilities for sex chromosomes, and partially because the complete fixation refers to one individual for the alternating backcross and two individuals for the father-daughter backcross.

Figure 5D and F show that the expected overall junction densities $\rho_{aa}^{mp}$ for the alternating backcross and the father-daughter backcross are lower than those of the RIL by sibling for autosomes and sex chromosomes, and that the junction densities for the alternating backcross are slightly smaller than those of the father-daughter backcross for autosomes. These results are consistent with those of Welsh and McMillan (2012): the number of chromosome segments in the final inbred lines increases from the alternating backcross to the father-daughter backcross and to the RIL by sibling.

### FGL exchangeability

We apply the recursive algorithm NON-EXCH to study the prior FGL exchangeability for the multi-way funnel breeding schemes (Figure 4). We assume that all founder parents are fully inbred, and assign FGLs from A to H to the eight inbred parents in order from left to right. To examine the FGL exchangeability, we calculate the ancestral coefficient $\varphi_{aa}^{mp}\left(ij\right)$ and the expected ancestral junction density $J_{aa}^{mp}\left(iijj\right),$ the latter being the only ancestral junction type for complete inbred individuals.

**Figure 6:**
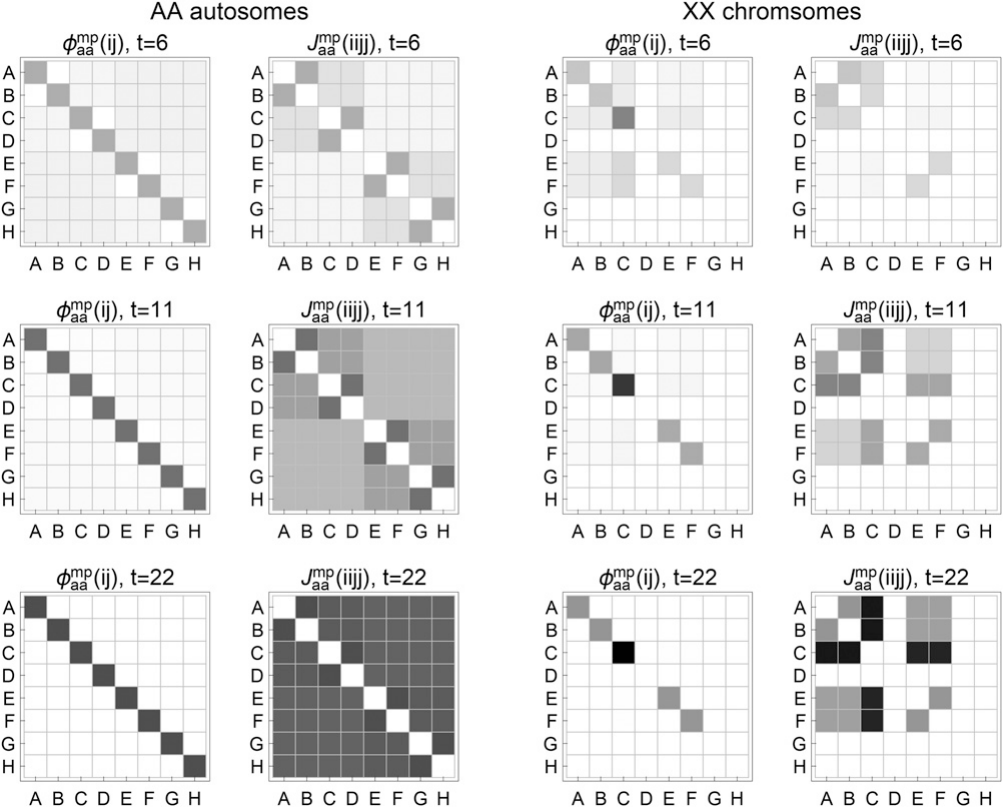
The FGL non-exchangeability patterns for the ancestral coefficient $\varphi_{aa}^{mp}\left(ij\right)$ and the expected ancestral junction density $J_{aa}^{mp}\left(iijj\right)$ in the eight-way RIL by sibling. The FGLs of eight founders are A-H from left to right.

Figure 6 shows the exchangeability in terms of $\varphi_{aa}^{mp}\left(ij\right)$ and $J_{aa}^{mp}\left(iijj\right)$ for the females in generation t=6, 11, and 22 for the RIL by sibling. The left panels show the results for autosomes. The FGL non-exchangeability confirms the pedigree inconsistency introduced by Liu *et al.* (2010): each of the four mating pairs of founder parents is impossible at a single locus in an individual, that is, $\varphi_{aa}^{mp}\left(ij\right)=0$ for (ji) or (ij)=(AB), (CD), (EF), and (GH). The FGL non-exchangeability shows a different pattern for $J_{aa}^{mp}\left(iijj\right):$ there are three levels of expected junction densities and the four mating pairs of founder parents have the highest values. For both $\varphi_{aa}^{mp}\left(ij\right)$ and $J_{aa}^{mp}\left(iijj\right),$ the non-exchangeability diminishes in generation 22 with almost complete inbreeding.

The right panels of Figure 6 show the exchangeability patterns for sex chromosomes of the RIL by sibling. The founder parents D, G, and H cannot pass their X chromosomes beyond F1, and thus their FGLs are impossible in generation t≥2. Similar to autosomes, $\varphi_{aa}^{mp}\left(ij\right)=0$ for (ji) or (ij)=(AB) and (EF). For $\varphi_{aa}^{mp}(ij)(i=j)$ or $\varphi_{a}^{m}\left(i\right),$ the probability of FGL C is around twice as large as that of A, B, E or F in generation t≥2, because the X chromosome carrying FGL C in generation 1 is inherited with probability 1, whereas each of the X chromosomes carrying FGLs A, B, E, and F in generation 1 is inherited with probability 1/2. Similarly, the values of $J_{aa}^{mp}\left(iijj\right)$ involving FGL C increase with inbreeding generation, they are about twice as large as others in generation 22.

**Figure 7:**
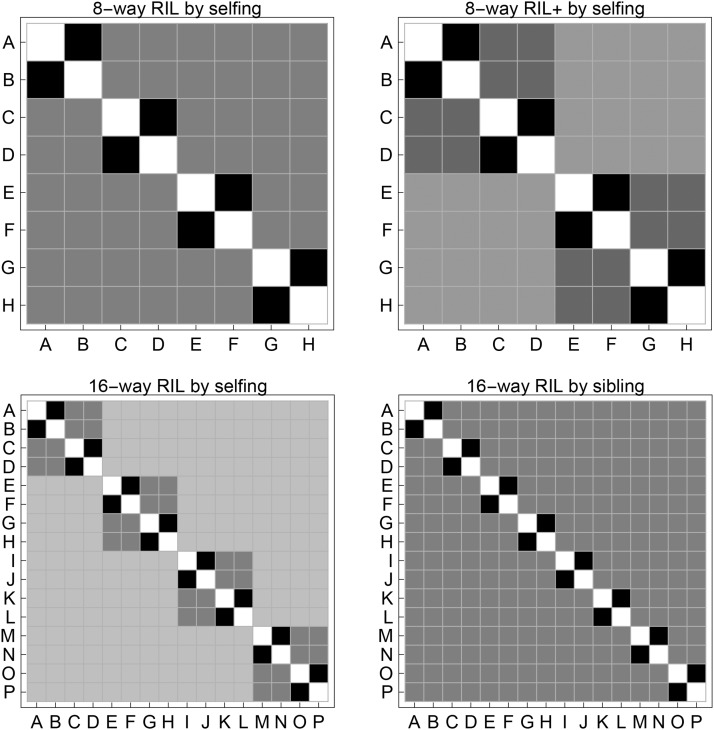
The FGL non-exchangeability patterns of the expected ancestral junction density $J_{aa}^{mp}\left(iijj\right).$ The results are for autosomes of the multi-way RIL by 100 generations of selfing or sibling. The FGLs for founder parents are letters starting from A up to P from left to right.

We examine the exchangeability patterns for the other multi-way funnel breeding schemes, and focus on autosomes since the FGL exchangeability does not hold in general for sex chromosomes because of the asymmetry between X and Y chromosomes. The non-exchangeability of $\varphi_{aa}^{mp}\left(ij\right)$ disappears in a completely inbred individual for all 4- 8- and 16-way breeding schemes, because of random chromosomal segregations over many inbreeding generations. However, the FGL exchangeability of $J_{aa}^{mp}\left(iijj\right)$ often does not hold even after for a completely inbred individual, for example, see Figure 7 for the breeding schemes after 100 generations of selfing inbreeding.

### Ancestral inference

Either one of the two recursive algorithms can be used as a prior to incorporate pedigree information for ancestral inference in multiparental populations from genotypic data, which will be illustrated as follows by simulated and real CC populations.

#### Simulated CC:

We simulate a CC population (Churchill *et al.* 2004) with 100 independent funnels, and take the female at the last generation. The SNP data for the eight founder mouse strains are from Collaborative Cross Consortium (2012), with marker density ∼5 SNPs per cM. The genotypic data of a sample female are obtained by simulating the FGLs forwardly and then combining them with the founder SNP data. Allelic errors are assumed to occur independently, and we simulate genotypic data of founders and sampled individuals with the same allelic error probability 0.005.

We analyze four simulated data sets: F11-AA, F11-XX, F22-AA, and F22-XX, where the first part denotes the generation, and the second part denotes the 19 pairs of autosomes (AA) or the sex chromosomes (XX). We perform two analyses for each data set by applying each of the two recursive algorithms as the prior for modeling ancestral origins along two homologous chromosomes within an individual. See Figure 6 for the prior distributions of $\varphi_{aa}^{mp}\left(ij\right)$ and $J_{aa}^{mp}\left(iijj\right)$ as a part of results obtained from the algorithm NON-EXCH. The true allelic error probability is used for the genotypic data of founders and sampled individuals.

Table 4 shows the evaluation of the prior FGL exchangeability on ancestral inference in the simulated CC population. The two analyses of the data set F22-AA are almost the same because there is approximately no prior FGL exchangeability for the autosomes in generation t=22, and the improvement of NON-EXCH over EXCH for F11-AA is ∼3% because of the FGL non-exchangeability such as that of $J_{aa}^{mp}\left(iijj\right)$ in Figure 6. The improvements for sex chromosomes are larger than those for autosomes because of the more pronounced FGL non-exchangeability (Figure 6). The improvement for the data set F22-XX is ∼6%, and it increases to ∼9% for F11-XX.

**Table 4:** Evaluation of the prior FGL exchangeability on ancestral inference in the simulated CC population consisting of 100 funnels

Probability	Simulated data set	EXCH	NON-EXCH	Improvement (%)*^a^*
Wrongly assigned*^b^*	F11-AA	0.02779	0.02715	2.3
	F11-XX	0.01821	0.01650	9.4
	F22-AA	0.02484	0.02476	0.3
	F22-XX	0.01540	0.01452	5.7
Wrongly called*^c^*	F11-AA	0.01962	0.01888	3.8
	F11-XX	0.01284	0.01175	8.5
	F22-AA	0.01766	0.01771	−0.3
	F22-XX	0.01170	0.01084	7.4

a The percentage decrease of the wrongly assigned (or called) probability for the analysis using the algorithm NON-EXCH, relative to the algorithm EXCH.

b One minus the posterior probability of being true ancestral states.

c One minus the fraction of called ancestral states being true ancestral states. At each SNP location within an individual, the ancestral state is called by its maximum posterior probability.

#### Real CC:

The real CC population consists of 120 lines that are sampled at generation *t* in the range of 8≤t≤14 (Durrant *et al.* 2011). For each line, we estimate the sampling generation by using the algorithm EXCH and taking the generation with the maximum likelihood, where the funnel code is not required. Then we estimate the funnel code by using the algorithm NON-EXCH and an iterative maximum likelihood, where a funnel code is proposed by slightly disturbing the current funnel code and it is accepted if the likelihood is increased. We compare the algorithms EXCH and NON-EXCH with GAIN (Liu *et al.* 2010), the latter being specifically developed for the CC.

To study the effect of marker densities on ancestral inference, we analyze only the first pair of homologous autosomes and thin the full dataset by taking every second SNP markers, and repeat to obtain nested sub datasets. The data fractions or the relative marker densities are given by $\rho_{M}=1,2^{-1},\text{...,}2^{-7}.$ The absolute maximum marker density is 145 SNPs per cM, ∼28 times higher than that of simulated CC. From the full dataset $\rho_{M}=1,$ we calculate the marginal posterior probabilities by NON-EXCH, EXCH, and GAIN, and set the pseudo-true values to the most probable ancestral origins if they are the same among the three methods. Overall the pseudo-true ancestral origins for 98.4% observed genotypes are obtained.

**Figure 8:**
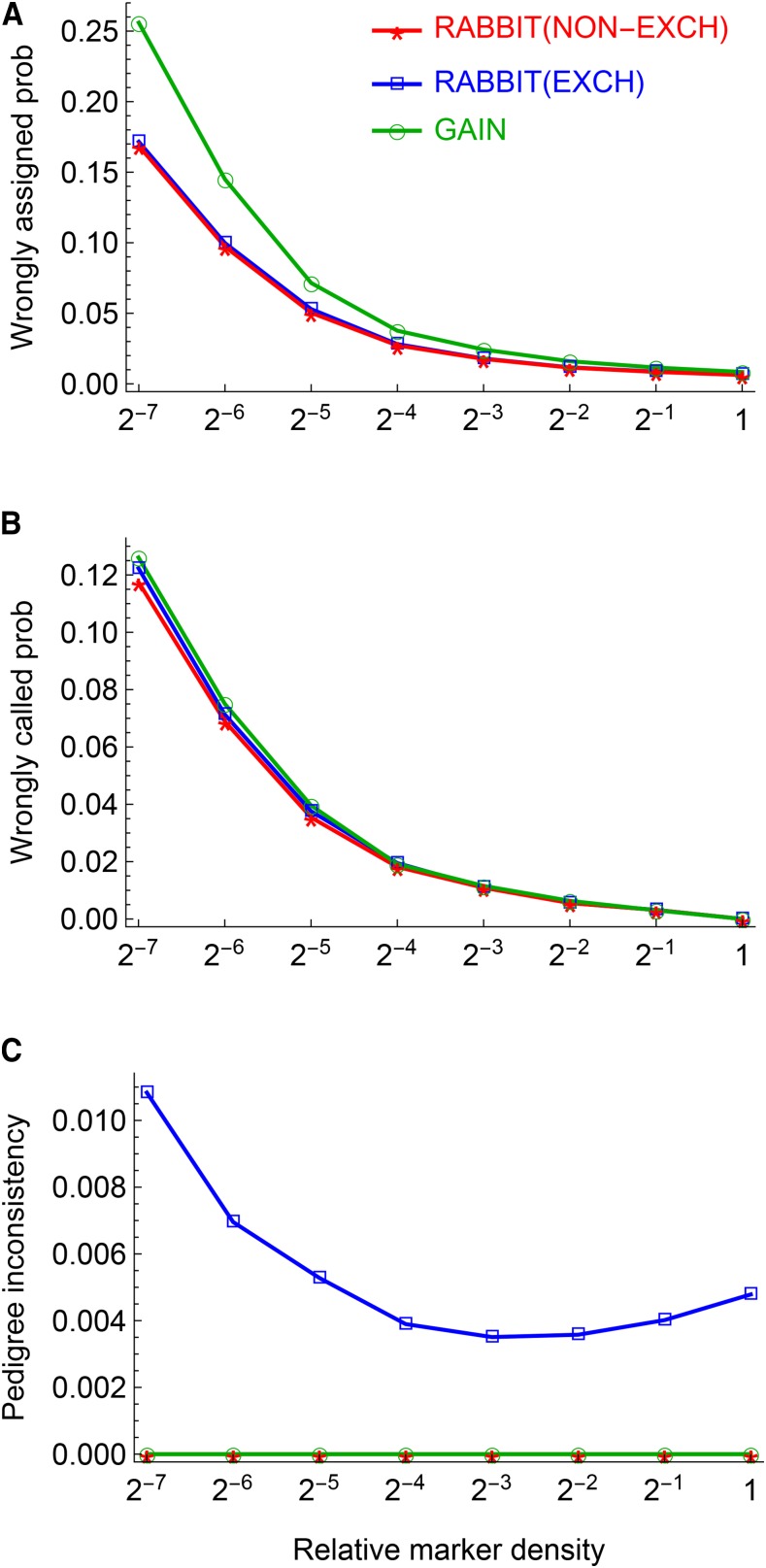
Evaluation of the recursive algorithms that are applied in the ancestral inference for the first pair of autosomes of the 120 real CC lines. Panels (A-C) show the results for the wrongly assigned probability, the wrongly called probability, and the pedigree inconsistency, respectively. The pedigree inconsistency is measured by the sum of the posterior probabilities over the four mating pairs of founder parents.

Figure 8 shows that the results on ancestral inferences are only slightly different among the three methods. The results of GAIN and NON-EXCH contain no pedigree inconsistencies, whereas NON-EXCH assigns ancestral origins to the impossible mating pairs of founder parents with probability around 0.005. The wrongly assigned probability for GAIN is larger than those of NON-EXCH and EXCH, and the difference increases with the decreasing marker density. The ranking of the wrong called probability from lowest to highest is NON-EXCH, EXCH, and GAIN, and the improvement of NON-EXCH over EXCH is around 4.2% and the improvement of NON-EXCH over GAIN is around 7.2% when relative density $\rho_{M}\geq 1/8.$ The similar performances between algorithms NON-EXCH and EXCH indicate that the prior FGL exchangeability is a reasonable approximation for autosomes in the CC.

## Discussion

We have developed two novel recursive algorithms for modeling genomic ancestral origins along two homologous chromosomes in a pedigree with arbitrary but known structure. The algorithms apply to both autosomes and sex chromosomes when they exist, and allow selfing in the pedigree. The extensive simulations on a real example pedigree show that the numerical results from the two recursive algorithms are consistent with the simulated results, apart from the stochasticity of gene flow. The Markov property is assumed for the ancestral origin process along two chromosomes, and thus genetic interference is assumed to be absent. The assumption does not affect the expected junction densities calculated from the two recursive algorithms, although it does affect the distribution of inter-junction distances including the variance of junction densities.

One important application of the recursive algorithms is to design a breeding scheme for accelerating the inbreeding process to obtain immortal lines and for maximizing the resolution of mapping QTL. Welsh and McMillan (2012) studied inbreeding processes among different types of multiparental crosses by simulations, and it takes approximately 5.5 hr to complete 100,000 simulations of eight-way RILs. In contrast, it takes less than one second for our recursive algorithms. Rockman and Kruglyak (2008) compared different intercross breeding designs in multiparental RILs by simulations, aiming at increasing the density of junctions (recombination breakpoints) and thus fine-mapping resolution. Our previous recursive algorithms (Zheng *et al.* 2014; Zheng 2015) can be used for calculating the junction density in random mating schemes, and the two new algorithms extend the calculation for any breeding schemes with fixed pedigrees. The two recursive algorithms can also be used in random mating schemes by applying them to many pedigrees that are simulated according to specified mating schemes and averaging the results, which would still require less number of replicates and less computational time than simulation studies.

The second important application of the two recursive algorithms is to provide an appropriate way of incorporating pedigree information for analyzing genotypic data in bi- or multiparental populations. Specifically, the two recursive algorithm can be used to calculate the process parameter values of hidden Markov models for genotypic data, that is, the prior probability distribution of ancestral origins (FGLs) at an initial site and the prior transition probability matrix describing how ancestral origins change along two homologous chromosomes within an individual. See Figure 1 of Zheng (2015) for an example. The application to the ancestral inference in the CC shows that the new algorithms implemented in RABBIT performs only slightly better than GAIN (Figure 8). However, GAIN is specifically designed for the CC, and our previous algorithm applies only to breeding schemes with stage-wise random mating. The new recursive algorithms have pronounced advantages of generality and computational efficiency, and they apply to arbitrary breeding pedigrees that are far away from random mating. For example, one of the multiparental barley populations consists of backcrossing, half-diallel crossing, and selfing, where one inbred founder is much stronger represented in the offspring lines (Liller *et al.* 2017).

Furthermore, we have applied the two recursive algorithms to incorporate pedigree information for genotype imputation in multiparental populations (Zheng *et al.* 2018), and the application for genetic linkage map construction in multiparental populations is under development.

## APPENDIX A: RECURRENCE RELATIONS OF ANCESTRAL COEFFICIENTS

We derive the recurrence relations of one-, two- and three-gene ancestral coefficients. For the one-gene ancestral coefficient $\varphi_{a}^{o}\left(i\right)$ of non-founder *a*, we have

$$\begin{array}{l} \varphi_{a}^{m}\left(i\right)=\frac{1}{2}\left[\varphi_{♀}^{m}\left(i\right)+\varphi_{♀}^{p}\left(i\right)\right],\\ \varphi_{a}^{p}\left(i\right)=\left\{\frac{1}{2}\left[\varphi_{♂}^{m}\left(i\right)+\varphi_{♂}^{p}\left(i\right)\right],\varphi_{♂}^{m}\left(i\right),\varphi_{♂}^{p}\left(i\right)\right\}. \end{array}$$

And for two-gene ancestral coefficient $\varphi_{ab}^{o_{1}o_{2}}\left(ij\right),$ we have

$$\varphi_{aa}^{oo}\left(ij\right)=1_{i=j}\varphi_{a}^{o}\left(i\right),\varphi_{aa}^{mp}\left(ij\right)=\varphi_{aa}^{pm}\left(ji\right)=\frac{1}{2}\left[\varphi_{a♀}^{pm}\left(ji\right)+\varphi_{a♀}^{pp}\left(ji\right)\right],\varphi_{ab}^{mo}\left(ij\right)=\frac{1}{2}\left[\varphi_{♀b}^{mo}\left(ij\right)+\varphi_{♀b}^{po}\left(ij\right)\right],a>b,\varphi_{ab}^{po}\left(ij\right)=\left\{\frac{1}{2}\left[\varphi_{♂b}^{mo}\left(ij\right)+\varphi_{♂b}^{po}\left(ij\right)\right],\varphi_{♂b}^{mo}\left(ij\right),\varphi_{♂b}^{po}\left(ij\right)\right\},a>b,\varphi_{ab}^{o_{1}o_{2}}\left(ij\right)=\varphi_{ba}^{o_{2}o_{1}}\left(ji\right),a<b,$$

for $o,o_{1},o_{2}\in \left\{m,p\right\},$ where the last equation is obtained by reversing the ordering of the two genes. See Figure 2A for a pictorial representation of the equation for $\varphi_{ab}^{mp}\left(ij\right).$

The symmetries such as $\varphi_{abc}^{o_{1}o_{2}o_{3}}\left(ijk\right)=\varphi_{acb}^{o_{1}o_{3}o_{2}}\left(ikj\right)=\ldots =\varphi_{cba}^{o_{3}o_{2}o_{1}}\left(kji\right)$ apply to three-gene ancestral coefficients by permuting the three genes, and thus we consider only a particular ordering a>b>c, and we have

$$\varphi_{aaa}^{ooo}\left(ijk\right)=\varphi_{a}^{o}\left(i\right)1_{i=j=k},\varphi_{aaa}^{o_{1}o_{1}o_{2}}\left(ijk\right)=\varphi_{aa}^{o_{1}o_{2}}\left(jk\right)1_{i=j},o_{1}\neq o_{2},\varphi_{aaa}^{o_{1}o_{2}o_{1}}\left(ijk\right)=\varphi_{aaa}^{o_{1}o_{1}o_{2}}\left(ikj\right),o_{1}\neq o_{2},\varphi_{aaa}^{o_{2}o_{1}o_{1}}\left(ijk\right)=\varphi_{aaa}^{o_{1}o_{1}o_{2}}\left(jki\right),o_{1}\neq o_{2},\varphi_{aab}^{o_{1}o_{1}o_{2}}\left(ijk\right)=\varphi_{ab}^{o_{1}o_{2}}\left(jk\right)1_{i=j},\varphi_{aab}^{mpo}\left(ijk\right)=\varphi_{aab}^{pmo}\left(jik\right)=\frac{1}{2}\left[\varphi_{a♀b}^{pmo}\left(jik\right)+\varphi_{a♀b}^{ppo}\left(jik\right)\right],\varphi_{abb}^{o_{1}o_{2}o_{2}}\left(ijk\right)=\varphi_{ab}^{o_{1}o_{2}}\left(ij\right)1_{j=k},\varphi_{abb}^{mo_{1}o_{2}}\left(ijk\right)=\frac{1}{2}\left[\varphi_{♀bb}^{mo_{1}o_{2}}\left(ijk\right)+\varphi_{♀bb}^{po_{1}o_{2}}\left(ijk\right)\right],o_{1}\neq o_{2},\varphi_{abb}^{po_{1}o_{2}}\left(ijk\right)=\left\{\frac{1}{2}\left[\varphi_{♂bb}^{mo_{1}o_{2}}\left(ijk\right)+\varphi_{♂bb}^{po_{1}o_{2}}\left(ijk\right)\right],\varphi_{♂bb}^{mo_{1}o_{2}}\left(ijk\right),\varphi_{♂bb}^{po_{1}o_{2}}\left(ijk\right)\right\},o_{1}\neq o_{2},\varphi_{abc}^{mo_{1}o_{2}}\left(ijk\right)=\frac{1}{2}\left[\varphi_{♀bc}^{mo_{1}o_{2}}\left(ijk\right)+\varphi_{♀bc}^{po_{1}o_{2}}\left(ijk\right)\right],\varphi_{abc}^{po_{1}o_{2}}\left(ijk\right)=\left\{\frac{1}{2}\left[\varphi_{♂bc}^{mo_{1}o_{2}}\left(ijk\right)+\varphi_{♂bc}^{po_{1}o_{2}}\left(ijk\right)\right],\varphi_{♂bc}^{mo_{1}o_{2}}\left(ijk\right),\varphi_{♂bc}^{po_{1}o_{2}}\left(ijk\right)\right\},$$

for $o,o_{1},o_{2}\in \left\{m,p\right\}.$

## APPENDIX B: RECURRENCE RELATIONS OF EXPECTED ANCESTRAL JUNCTION DENSITIES

The joint recurrence relations of $J_{ab}^{o_{1}o_{2}}\left(ijkj\right)$ and $J_{ab}^{o_{1}o_{2}}\left(ijik\right)$ are the same as those of $J_{ab}^{o_{1}o_{2}}\left(1232\right)$ and $J_{ab}^{o_{1}o_{2}}\left(1213\right),$ except that identity states are replaced by ancestral states and that the contributions of three-gene ancestral coefficients account for the asymmetry of FGLs. We have

$$J_{ab}^{mo}\left(ijkj\right)=\frac{1}{2}\left[J_{♀b}^{mo}\left(ijkj\right)+J_{♀b}^{po}\left(ijkj\right)\right]0_{a=b}^{m=o}+\frac{1}{2}\left[\varphi_{\mathrm{♀♀}b}^{mpo}\left(ikj\right)+\varphi_{\mathrm{♀♀}b}^{pmo}\left(ikj\right)\right]1_{i\neq j\neq k}0_{a=b}^{m=o},a\geq b,J_{ab}^{po}\left(ijkj\right)=\{\frac{1}{2}\left[J_{♂b}^{mo}(ijkj)+J_{♂b}^{po}(ijkj)\right]+\frac{1}{2}\left[\varphi_{\mathrm{♂♂}b}^{mpo}(ikj)+\varphi_{\mathrm{♂♂}b}^{pmo}(ikj)\right]1_{i\neq j\neq k},J_{♂b}^{mo}(ijkj),J_{♂b}^{po}(ijkj)\}0_{a=b}^{p=o},a\geq b,J_{ab}^{o_{1}o_{2}}\left(ijkj\right)=J_{ba}^{o_{2}o_{1}}\left(jijk\right),a<b,J_{ab}^{mo}\left(ijik\right)=\frac{1}{2}\left[J_{♀b}^{mo}\left(ijik\right)+J_{♀b}^{po}\left(ijik\right)\right]0_{a=b}^{m=o},a\geq b,J_{ab}^{po}\left(ijik\right)=\left\{\frac{1}{2}\left[J_{♂b}^{mo}\left(ijik\right)+J_{♂b}^{po}\left(ijik\right)\right],J_{♂b}^{mo}\left(ijik\right),J_{♂b}^{po}\left(ijik\right)\right\}0_{a=b}^{p=o},a\geq b,J_{ab}^{o_{1}o_{2}}\left(ijik\right)=J_{ba}^{o_{2}o_{1}}\left(jiki\right),a<b,$$

for $o,o_{1},o_{2}\in \left\{m,p\right\},$ where $J_{ba}^{o_{2}o_{1}}\left(jijk\right)$ in the 3rd equation and $J_{ba}^{o_{2}o_{1}}\left(jiki\right)$ in the 6th equation can be transformed into the form of $J_{ba}^{o_{2}o_{1}}\left(ijik\right)$ and $J_{ba}^{o_{2}o_{1}}\left(ijkj\right),$ respectively, by swapping *i* and *j*. There are no contributions of the three-gene ancestral coefficients in the equations for $J_{ab}^{mo}\left(ijik\right)$ and $J_{ab}^{po}\left(ijik\right)$ because crossovers between the two parental haplotypes of the tracing haplotype within individual *a* are invisible. See Figure 2B for a pictorial representation of the equation for $J_{ab}^{mp}\left(ijkj\right).$

The joint recurrence relations of $J_{ab}^{o_{1}o_{2}}\left(ijjj\right)$ and $J_{ab}^{o_{1}o_{2}}\left(ijii\right)$ are the same to those of $J_{ab}^{o_{1}o_{2}}\left(ijkj\right)$ and $J_{ab}^{o_{1}o_{2}}\left(ijik\right),$ except that the contributions of the three-gene ancestral coefficients have slightly different forms. We have

$$J_{ab}^{mo}\left(ijjj\right)=\frac{1}{2}\left[J_{♀b}^{mo}\left(ijjj\right)+J_{♀b}^{po}\left(ijjj\right)\right]0_{a=b}^{m=o}+\frac{1}{2}\left[\varphi_{\mathrm{♀♀}b}^{mpo}\left(ijj\right)+\varphi_{\mathrm{♀♀}b}^{pmo}\left(ijj\right)\right]1_{i\neq j}0_{a=b}^{m=o},a\geq b,J_{ab}^{po}\left(ijjj\right)=\{\frac{1}{2}\left[J_{♂b}^{mo}(ijjj)+J_{♂b}^{po}(ijjj)\right]+\frac{1}{2}\left[\varphi_{\mathrm{♂♂}b}^{mpo}(ijj)+\varphi_{\mathrm{♂♂}b}^{pmo}(ijj)\right]1_{i\neq j},J_{♂b}^{mo}(ijjj),J_{♂b}^{po}(ijjj)\}0_{a=b}^{p=o},a\geq b,J_{ab}^{o_{1}o_{2}}\left(ijjj\right)=J_{ba}^{o_{2}o_{1}}\left(jijj\right),a<b,J_{ab}^{mo}\left(ijii\right)\;=\frac{1}{2}\left[J_{♀b}^{mo}\left(ijii\right)+J_{♀b}^{po}\left(ijii\right)\right]\;0_{a=b}^{m=o},a\geq b,J_{ab}^{po}\left(ijii\right)=\left\{\frac{1}{2}\left[J_{♂b}^{mo}\left(ijii\right)+J_{♂b}^{po}\left(ijii\right)\right],J_{♂b}^{mo}\left(ijii\right),J_{♂b}^{po}\left(ijii\right)\right\}0_{a=b}^{p=o},a\geq b,J_{ab}^{o_{1}o_{2}}\left(ijii\right)=J_{ba}^{o_{2}o_{1}}\left(jiii\right),a<b,$$

for $o,o_{1},o_{2}\in \left\{m,p\right\},$ where $J_{ba}^{o_{2}o_{1}}\left(jijj\right)$ in the 3rd equation and $J_{ba}^{o_{2}o_{1}}\left(jiii\right)$ in the 6th equation can be transformed into the form of $J_{ba}^{o_{2}o_{1}}\left(ijii\right)$ and $J_{ba}^{o_{2}o_{1}}\left(ijjj\right)$, respectively, by swapping *i* and *j*.

## References

[bib1] Baird S. Barton N. Etheridge A., 2003 The distribution of surviving blocks of an ancestral genome. Theor. Popul. Biol. 64: 451–471. 10.1016/S0040-5809(03)00098-4 14630482

[bib2] Bauman L. E. Sinsheimer J. S. Sobel E. M. Lange K., 2008 Mixed effects models for quantitative trait loci mapping with inbred strains. Genetics 180: 1743–1761. 10.1534/genetics.108.091058 18791243 PMC2581972

[bib3] Bennett J. H., 1953 Junctions in inbreeding. Genetica 26: 392–406. 10.1007/BF01690623 13142314

[bib4] Bennett J. H., 1954 The distribution of heterogeneity upon inbreeding. J. R. Stat. Soc. Series B Stat. Methodol. 16: 88–99.

[bib5] Bickeböller H. Thompson E. A., 1996 Distribution of genome shared ibd by half-sibs: approximation by the poisson clumping heuristic. Theor. Popul. Biol. 50: 66–90. 10.1006/tpbi.1996.0023 8776838

[bib6] Broman K. W., 2012 Genotype probabilities at intermediate generations in the construction of recombinant inbred lines. Genetics 190: 403–412. 10.1534/genetics.111.132647 22345609 PMC3276635

[bib7] Chapman A. Jacquard A., 1971 Un isolat d’Amerique centrale: les indiens Jicaques du Honduras, Génétique et Populations, Presses Universitaires de France, Paris.

[bib8] Chapman N. Thompson E., 2003 A model for the length of tracts of identity by descent in finite random mating populations. Theor. Popul. Biol. 64: 141–150. 10.1016/S0040-5809(03)00071-6 12948676

[bib9] Churchill G. A. Airey D. C. Allayee H. Angel J. M. Attie A. D., 2004 The collaborative cross, a community resource for the genetic analysis of complex traits. Nat. Genet. 36: 1133–1137. 10.1038/ng1104-1133 15514660

[bib10] Cockerham C. C. Weir B. S., 1973 Descent measures for 2 loci with some applications. Theor. Popul. Biol. 4: 300–330. 10.1016/0040-5809(73)90013-0 4747657

[bib20] Collaborative Cross Consortium, 2012 The genome architecture of the collaborative cross mouse genetic reference population. Genetics 190: 389–401. 10.1534/genetics.111.132639 22345608 PMC3276630

[bib11] Denniston C., 1974 Extension of probability approach to genetic relationship - one locus. Theor. Popul. Biol. 6: 58–75. 10.1016/0040-5809(74)90031-8 4425405

[bib12] Donnelly K. P., 1983 The probability that related individuals share some section of genome identical by descent. Theor. Popul. Biol. 23: 34–63. 10.1016/0040-5809(83)90004-7 6857549

[bib13] Durrant C. Tayem H. Yalcin B. Cleak J. Goodstadt L., 2011 Collaborative cross mice and their power to map host susceptibility to aspergillus fumigatus infection. Genome Res. 21: 1239–1248. 10.1101/gr.118786.110 21493779 PMC3149491

[bib14] Fisher R., 1949 The Theory of Inbreeding, Oliver and Boyd, London.

[bib15] Fisher R., 1954 A fuller theory of junctions in inbreeding. Heredity 8: 187–197. 10.1038/hdy.1954.17

[bib16] Garcia-Cortes L. A., 2015 A novel recursive algorithm for the calculation of the detailed identity coefficients. Genet. Sel. Evol. 47: 33. 25926309 10.1186/s12711-015-0108-6 PMC4414392

[bib17] Haldane J. Waddington C., 1931 Inbreeding and linkage. Genetics 16: 357–374. 17246626 10.1093/genetics/16.4.357 PMC1201105

[bib18] Hill W. G. Weir B. S., 2007 Prediction of multi-locus inbreeding coefficients and relation to linkage disequilibrium in random mating populations. Theor. Popul. Biol. 72: 179–185. 10.1016/j.tpb.2006.05.006 17575994 PMC2729754

[bib19] Huang B. E. George A. W. Forrest K. L. Kilian A. Hayden M. J., 2012 A multiparent advanced generation inter-cross population for genetic analysis in wheat. Plant Biotechnol. J. 10: 826–839. 10.1111/j.1467-7652.2012.00702.x 22594629

[bib21] Jacquard A., 1974 The Genetic Structure of Populations, Springer-Verlag, New York 10.1007/978-3-642-88415-3

[bib22] Johannes F. Colome-Tatche M., 2011 Quantitative epigenetics through epigenomic perturbation of isogenic lines. Genetics 188: 215–227. 10.1534/genetics.111.127118 21385727 PMC3120148

[bib23] Karigl G., 1981 A recursive algorithm for the calculation of identity coefficients. Ann. Hum. Genet. 45: 299–305. 10.1111/j.1469-1809.1981.tb00341.x 7305283

[bib24] Kover P. X. Valdar W. Trakalo J. Scarcelli N. Ehrenreich I. M., 2009 A multiparent advanced generation inter-cross to fine-map quantitative traits in *Arabidopsis thaliana*. PLoS Genet. 5: e1000551 10.1371/journal.pgen.1000551 19593375 PMC2700969

[bib25] Li C. H. Li Y. X. Bradbury P. J. Wu X. Shi Y. S., 2015 Construction of high-quality recombination maps with low-coverage genomic sequencing for joint linkage analysis in maize. BMC Biol. 13: 78 10.1186/s12915-015-0187-4 26390990 PMC4578237

[bib26] Liller C. B. Walla A. Boer M. P. Hedley P. Macaulay M., 2017 Fine mapping of a major QTL for awn length in barley using a multiparent mapping population. Theor. Appl. Genet. 130: 269–281. 10.1007/s00122-016-2807-y 27734096 PMC5263209

[bib27] Liu E. Y. Zhang Q. McMillan L. Pardo-Manuel de Villena F. Wang W., 2010 Efficient genome ancestry inference in complex pedigrees with inbreeding. Bioinformatics 26: i199–i207. 10.1093/bioinformatics/btq187 20529906 PMC2881372

[bib28] Mackay I. J. Bansept-Basler P. Barber T. Bentley A. R. Cockram J., 2014 An eight-parent multiparent advanced generation inter-cross population for winter-sown wheat: creation, properties, and validation. G3 (Bethesda) 4: 1603–1610. 10.1534/g3.114.012963 25237112 PMC4169152

[bib29] MacLeod A. K. Haley C. S. Woolliams J. A., 2005 Marker densities and the mapping of ancestral junctions. Genet. Res. 85: 69–79. 10.1017/S0016672305007329 16089037

[bib30] Martin O. C. Hospital F., 2011 Distribution of parental genome blocks in recombinant inbred lines. Genetics 189: 645–654. 10.1534/genetics.111.129700 21840856 PMC3189807

[bib31] Nadot R. Vayssiex G., 1973 Algorithme du calcul des coefficients d’identite. Biometrics 29: 347–359. 10.2307/2529397

[bib32] Pascual L. Desplat N. Huang B. E. Desgroux A. Bruguier L., 2015 Potential of a tomato magic population to decipher the genetic control of quantitative traits and detect causal variants in the resequencing era. Plant Biotechnol. J. 13: 565–577. 10.1111/pbi.12282 25382275

[bib33] Rockman M. V. Kruglyak L., 2008 Breeding designs for recombinant inbred advanced intercross lines. Genetics 179: 1069–1078. 10.1534/genetics.107.083873 18505881 PMC2429860

[bib34] Rostron J., 1978 Computation of inbreeding coefficients. Ann. Hum. Genet. 41: 469–475. 10.1111/j.1469-1809.1978.tb00918.x 655636

[bib35] Sannemann W. Huang B. E. Mathew B. Leon J., 2015 Multi-parent advanced generation inter-cross in barley: high-resolution quantitative trait locus mapping for flowering time as a proof of concept. Mol. Breed. 35: 86 10.1007/s11032-015-0284-7

[bib36] Stam P., 1980 The distribution of the fraction of the genome identical by descent in finite random mating populations. Genet. Res. 35: 131–155. 10.1017/S0016672300014002

[bib37] Stefanov V., 2000 Distribution of genome shared identical by descent by two individuals in grandparent-type relationship. Genetics 156: 1403–1410. 11063711 10.1093/genetics/156.3.1403 PMC1461320

[bib38] Teuscher F. Broman K. W., 2007 Haplotype probabilities for multiple-strain recombinant inbred lines. Genetics 175: 1267–1274. 10.1534/genetics.106.064063 17151250 PMC1840090

[bib39] Thompson E. A., 1983 A recursive algorithm for inferring gene origins. Ann. Hum. Genet. 47: 143–152. 10.1111/j.1469-1809.1983.tb00981.x 6881911

[bib40] Thompson E. A., 1988 2-locus and 3-locus gene identity by descent in pedigrees. IMA J. Math. Appl. Med. Biol. 5: 261–279. 10.1093/imammb/5.4.261 3241098

[bib41] Weir B. S. Cockerham C. C., 1969 Pedigree mating with 2 linked loci. Genetics 61: 923–940. 5364970 10.1093/genetics/61.4.923 PMC1212252

[bib42] Welsh C. E. McMillan L., 2012 Accelerating the inbreeding of multi-parental recombinant inbred lines generated by sibling matings. G3 (Bethesda) 2: 191–198. 10.1534/g3.111.001784 22384397 PMC3284326

[bib43] Xu S. Z., 2013 Genetic mapping and genomic selection using recombination breakpoint data. Genetics 195: 1103–1115. 10.1534/genetics.113.155309 23979575 PMC3813840

[bib44] Zheng C., 2015 Modeling X-linked-linked ancestral origins in multiparental populations. G3 (Bethesda) 5: 777–801. 10.1534/g3.114.016154 25740936 PMC4426366

[bib45] Zheng C. Boer M. P. van Eeuwijk F. A., 2014 A general modeling framework for genome ancestral origins in multiparental populations. Genetics 198: 87–101. 10.1534/genetics.114.163006 25236451 PMC4174956

[bib46] Zheng C. Boer M. P. van Eeuwijk F. A., 2018 Accurate genotype imputation in multiparental populations from low-coverage sequence. Genetics genetics.300885.2018 10.1534/genetics.118.300885 PMC6116951 30045858

[bib47] Zhou J. J. Ghazalpour A. Sobel E. M. Sinsheimer J. S. Lange K., 2012 Quantitative trait loci association mapping by imputation of strain origins in multifounder crosses. Genetics 190: 459–473. 10.1534/genetics.111.135095 22143921 PMC3276647

